# Efficacy of Peer-to-Peer Education for Emergency Medicine Resident Financial Literacy: Curriculum Development Study

**DOI:** 10.7759/cureus.32668

**Published:** 2022-12-18

**Authors:** Scott L Rupp, Claire Abramoff, Kristin McCloskey

**Affiliations:** 1 Emergency Medicine, Einstein Medical Center Philadelphia, Philadelphia, USA; 2 Emergency Medicine/Medical Toxicology, Einstein Medical Center Philadelphia, Philadelphia, USA

**Keywords:** emergency medicine, quantitative improvement, medical education, financial literacy, curriculum development

## Abstract

Background: Early career physicians are under enormous stress from rigorous academic demands and financial insecurity due to increasingly large loan burdens and stagnant income. There are no institutionally required training programs to educate professionals on financial pitfalls or strategies for overcoming these burdens. Fiscal ignorance leaves them in a vulnerable position to be taken advantage of often, at great personal cost.

Methods: Using a cross-sectional, convenience sample of emergency medicine residents at a single center, we evaluated the benefit of a six-month curriculum on financial education. Focusing on topics that were most pertinent early in medical careers, we assessed the utility of a six-lecture series totaling three hours of education on fundamental financial literacy. Lectures were given by a single educator with no formal financial background in the following areas: general principles, student loans, retirement accounts, basic taxes, real estate, and insurance.

Results: Using pre-test and post-test information on 55 residents, financial literacy, as assessed by a 24-question multiple-choice survey, increased from 50% to 62% (p=<.001). Subgroup analysis was also performed within each financial category as well as by postgraduate year (PGY) of training. Raw data of individual percentages achieving specific financial milestones demonstrated an objective increase in the number of residents contributing to retirement accounts, creating an emergency fund, and establishing student loan repayment plans after the curriculum.

Conclusions: Programs can institute sufficient financial literacy education for trainees that covers financial foundations. These programs can be taught without financial professional expertise or long hours of didactics.

## Introduction

Resident physicians are given an insurmountable task - the comprehension of science to create the art of medicine. This encompasses not only the objective of acquiring all the medical information that they will use to treat their patients but also learning how and when to best apply it. This monumental task occurs all while facing crippling debt, social withdrawal, and sleep deprivation. After completing medical school and frequently acquiring upward of $220,000 in student loan debt [1], residents are left to their own devices to learn to deal with this debt. The conventional thought is that most physicians will end up making a significant salary and have minimum difficulty tackling repayment - despite evidence of wage stagnation and impending concern about future job prospects in Emergency Medicine (EM) [2]. There is no standard approach or institutional programs required by the American College of Graduate Medical Education (ACGME) to help prepare residents for these burgeoning financial difficulties [3] - though non-medical adjunct education is certainly within its authority to require [4]. As part of the residency, institutions provide education outside the spectrum of typical medical topics: fatigue, equal opportunity, and burnout resilience. However well-intentioned, institutions often fall short of providing objective means to deal with burnout.

Burnout is a common impairment amongst healthcare professionals across all clinical specialties, and it has only worsened amidst the worldwide coronavirus pandemic. Rising patient demands, decreased purchasing power [5], and significant job loss have all demanded more from the healthcare field. Previous studies have identified that 33%-65% of providers are subject to burnout [6,7]. Residents are required to be educated and assessed for signs of burnout as required by the ACGME [4], but they are often inadequately provided with the tools to combat this known stressor. This is demonstrated by persistently high burnout levels despite the 2017 ACGME revision to address well-being more directly and comprehensively. Burnout is a dynamic threat but commonly understood as a "moral injury" where the expectations of healthcare as a mission fail to meet reality. Although the struggle against burnout is multi-faceted, providing and ensuring financial literacy is a core tenant to prolonging the longevity of a career and overall job satisfaction [8,9].

In a typical business model, increasing profits come from trial and error, which yields progressively improved financial acumen and, in turn, increases income. Physicians, by contrast, come into their large incomes suddenly regardless of financial literacy. Despite years to practice in their field, attendings are often inept at wealth management despite finding themselves with heavy economic burdens and expectations [10]. Understanding that the average resident makes approximately $64,000 a year [11], they move from a position that may be paid $17-22 per hour one year to four or five times that the next year. This income imbalance creates risk - predatory wealth managers, and financial advisors can pay large sums to have the undivided attention of young physicians [12]. When well-meaning hospital administrators do recognize the need for financial education, they often turn to a financial advisor or local firm to educate and assist their staff. However, while the advice contained within these presentations is factual, it is seldom without bias [13]. Financial advisors and firms are in the business of selling financial advice and services, which inevitably creates an undertone for the financial education that they provide. Unless an advisor operates under a fiduciary obligation, presentations may contain ill-directed advice and intimidating figures and are commonly used to create a sense of need for their products - regardless of whether the product or service is in the client’s best interest [14].

It is difficult to find objective, competent financial education outside of a university setting. In a study of barriers to financial education, 80% of respondents identified the “inability to find an educator” as an obstacle to curriculum change [6]. Beyond being required to overcome the American taboo of discussing personal financial figures, they are often not given the time required to make an impact. Take, for example, a typical residency program that may allocate a one-hour time slot once every year or so. During that time, the speaker is expected to provide all relevant financial advice to a large group of residents indifferent of what stage of life they may be in: newly graduated from medical school, current homeowners, parents, or dual-income families are all discussed in the same tone [15]. This leaves little room for more than anecdotes and “rules-of-thumb”, which at best, inspire further investigation and, at worst - overwhelm the learner entirely. Newly aware of the metaphorical financial tightrope they find themselves on, residents begin to suffer paralysis by analysis or are left feeling despaired entirely. Insufficient education undercuts the years of service that it takes to achieve such a lofted position, and the resources provided by the American Association of Medical Colleges (AAMC) are insufficient [16]. Despite compensation being the spine of the healthcare field, there is a paucity of data surrounding physician education and the long-term effects of career satisfaction [17].

## Materials and methods

Study design and population

A single-center, four-year emergency medicine program of 60 physician residents and two physician assistants (PA) in Philadelphia, PA, were exposed to six months of a guided financial education curriculum during their routine didactic sessions. These didactic sessions were slide-based and administered over virtual conference software or in person. There were no handouts or adjuncts otherwise provided, but residents were given time to ask questions or email clarifying questions. Although the weekly conference is required, attendance is typically around 80% due to shift schedules and off-service conflicts. Although not explicitly included in the study design, medical students and faculty were also present at these sessions and were freely encouraged to participate and provide feedback or experience. Credentials for the resident educator include 11 years of financial investing experience, approximately 350 hours of open-access financial literacy education, personal management of a six-figure portfolio, and several books on personal financial education. 

Before all lectures, a baseline 24-question multiple-choice survey (Appendix 1), similar to well-validated financial literacy assessments [18] was sent to the residency (Table 1). Participation was highly encouraged but not mandatory. These questions were composed by the resident educator providing the lectures and were objective answers from the lecture material, specifically, approximately three to five questions per lecture. Many, although not all, of these questions, were reviewed in a three-question review after each one of the lectures. Six months after the initial survey and after the final lecture, a 25-question post-curriculum multiple-choice quiz was sent to those that responded to the initial survey. Many of the questions on the post-test were changed from the original but were all derived from the material discussed in the lecture slides.

**Table 1:** Demographic information and academic year (AY) for participants PGY: Post-graduate year

Table 1. Baseline Characteristics	
Characteristic	Subjects (n=55)
	no. (%)
Sex	
Male	36 (65)
Female	19 (35)
Race	
White	43 (78)
Asian	9 (16)
Black	2 (4)
Other	1 (2)
Training Year	
PGY-1	16 (29)
PGY-2	15 (27)
PGY-3	12 (22)
PGY-4	12 (22)

Quiz results were collected using Microsoft Office Forms and were not anonymous to the reviewing author to allow for matching pre/post-curriculum. Participants were not given their test results on either exam unless requested and were blinded to all other test-taker results. Residents were not encouraged or explicitly prohibited from using outside material while taking the quiz or required to take it in one sitting. The approximate time to complete the pre-curriculum and post-curriculum survey were eight and nine minutes, respectively. The project was approved as Institutional Review Board (IRB) exempt per the institution.

Outcomes and variables

The outcome in question was quiz performance before and after the curriculum assessing six areas of financial literacy: general principles, student loan repayment programs and public student loan forgiveness (PSLF), common retirement or savings accounts, introductory taxes, common physician insurance products, and an introduction to real estate investing. Additionally, after the survey, residents were asked to identify which financial “milestones” they had completed before any education. The battery of these milestones included: opening an individual retirement account (IRA), contributing to an employer 401(k)/403(b), obtaining disability insurance, creating a personalized financial plan, establishing an emergency fund, enrolling in a student loan repayment plan, paying off all outstanding credit card or high-interest debt, purchasing term life insurance, opening a personal brokerage account, becoming a homeowner, and paying off all outstanding student loan balance. The percentage of residents self-reporting that they have accomplished each milestone was reported as a gross percentage pre/post-curriculum without statistical analysis.

Statistical analysis

Standard descriptive summary statistics were used to characterize the subject demographics and responses. Paired-samples t-tests were used to compare mean scores at baseline and after undergoing the curriculum. Confidence intervals (95%) were derived from standard errors and reported from significant results. Subgroup analysis was also completed using t-tests. Financial milestones were expressed as absolute percentages of the total cohort, not as a relative increase from the baseline.

## Results

**Figure 1:**
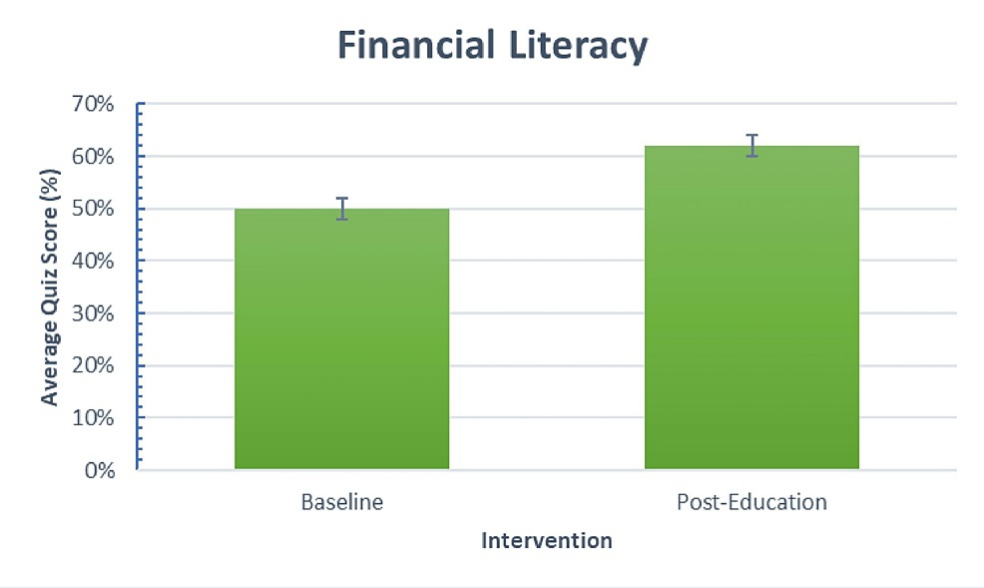
Pre- and post-intervention comparison for overall average on financial literacy quiz. Error bars calculated as standard error (2%)

The results were collected from 55 participating residents on the pre-curriculum survey and 53 completing the post-curriculum survey. Two participants did not complete their post-curriculum survey and were not included in the statistical analysis. Age and demographic information were not requested from participants for the survey (Table 1). Participants were 65% male and primarily Caucasian. The primary outcome, as demonstrated by quiz score, improved from 50% (SD 10%; 95% CI 47-53%) on baseline testing, consistent with percentages demonstrated in multiple prior studies [3,19], to 62% (SD 16%; 95% CI 58%-66%), p-value < .001 after intervention (Figure 1). Participants reported attending four of six lectures on average (range 1-6).

Results were broken down by academic year (PGY1-4) and financial education curriculum topics: Principles, student loans, retirement accounts, taxes, insurance, and real estate. Across the sub-groups, baseline understanding of topics ranged from 25%-64% (Table 2). Post-curriculum scores ranged from 49%-76%, and the only category to have a statistically significant improvement from baseline was insurance (Table 2). Within various PGY classes, baseline understanding was largely homogenous, 48% to 53% (Table 3). All classes improved in aggregate, 57% to 68%, demonstrating a statistically significant increase across every class (Table 3).

**Table 2:** Pre- and post-intervention quiz average comparison for each financial category

Sub Group Analysis						
Principles	Student Loans	Retirement	Taxes	Insurance	Real Estate	
64%	25%	50%	42%	61%	38%	Pre-Test
64%	57%	65%	49%	76%	60%	Post- Test
0.946	0.064	0.084	0.386	0.006	0.096	p-value

**Table 3:** Pre- and post-curriculum quiz scores by class

Class Year				
PGY-1	PGY-2	PGY-3	PGY-4	% (no. )
48% (16)	49% (15)	53% (12)	50% (12)	Pre-Test
62% (14)	57% (15)	68% (12)	63% (12)	Post- Test
0.010	0.046	0.001	0.002	p-value

During the six months that the study was taking place, there was an 11% absolute increase in people who opened an IRA. There was a 10% increase in people who were consciously contributing to an employer-sponsored retirement account. 14% of people newly developed a financial plan, and 10% newly created an emergency fund. 15% enrolled in a specific student loan repayment plan or refinance. 12% of people purchased term life insurance. There was a modest increase in people who purchased disability insurance and paid off high-interest credit card debt. There were no observable changes in the homeowners or those reporting that they paid off their student loans.

## Discussion

This study demonstrates the utility and feasibility of instituting a curriculum of financial education during residency. It is clearly possible to make an objective, meaningful change in financial literacy to their benefit and to support the field of healthcare. The education that is commonly available and received is either insufficient to properly educate the important topics to residents or comes from a person who has a direct conflict of interest. The lessons in this curriculum are not secret - they are simple lessons that are typically learned over a physician’s career. The longer it takes to learn the foundations of financial literacy, the larger the effect of mistakes that could potentially lead to years of lost revenue.

Understanding that financial independence (FI), i.e., the ability to cover all financial obligations with the proceeds of currently owned assets with no requirement of earned income, is the ultimate objective in mind [20]. The study quantified several financial milestones as surrogate markers of progression to FI. Although not all these milestones are required to reach FI, each signifies an objective measure of progress.

**Figure 2:**
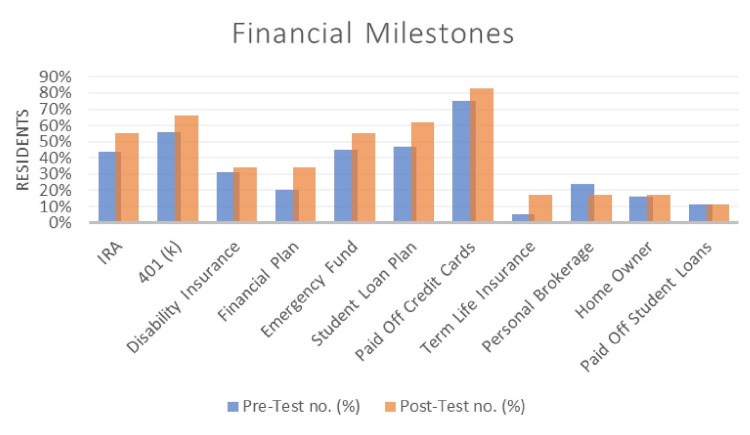
Pre- and post-intervention comparison for financial milestones that residents self-reported

Although the statistical difference between the financial literacy quizzes was 12%, the practical significance is seen in Table 4. The differences seen before and after the curriculum were consistently positive. The general assumption is that financial lessons are learned passively over the years but observing the pre-curriculum quiz values between classes suggests otherwise. There is no difference between financial literacy at baseline, and most classes showed similar improvement, which is contradictory to improved knowledge in more advanced PGY years seen in other studies [21]. There is slightly increased participation from PGY-1s in comparison to peer groups, which may explain the below-average understanding of student loan programs since the education was initiated at the start of the academic year. The discrepancies seen in significant differences between Figure 1 and Figure 2 may be due to knowledge atrophy over the six-month curriculum, whereas acquiring insurance or opening a retirement account may only take a few minutes.

**Table 4:** Pre- and post-intervention comparison for self-reported resident financial milestones IRA: Individual retirement accounts

	Pre-Test no. (%)	Post-Test no. (%)
IRA	24 (44%)	29 (55%)
401 (k)	31 (56%)	35 (66%)
Disability Insurance	17 (31%)	18 (34%)
Financial Plan	11 (20%)	18 (34%)
Emergency Fund	25 (45%)	29 (55%)
Student Loan Plan	26 (47%)	33 (62%)
Paid Off Credit Cards	41 (75%)	44 (83%)
Term Life Insurance	3 (5%)	9 (17%)
Personal Brokerage	13 (24%)	9 (17%)
Home Owner	9 (16%)	9 (16%)
Paid Off Student Loans	6 (11%)	6 (11%)

Limitations

There are numerous limitations to this study, including attendance, researcher bias with the absence of blinding, generalizability, replication, and the absence of longitudinal data. Despite conferences being a required activity, the mean number of lectures attended by residents was four of the six offered. Individual, real-time lecture attendance was not recorded, and the results were self-reported on the post-curriculum survey. People may not have attended lectures they had no interest in or may have been less engaged with the material while lectures were held over video conferences. Alternatively, the results may have a positive skew due to a lack of blinding between the statistical analysis and the educator's objectives. Many of the questions on the quiz were recycled from the previous quiz six months prior, and it is possible that after seeing the questions, students looked up the answer. The quizzes were taken in good faith and were not explicitly told to refrain from outside material help or collaboration. 

The education in this curriculum was provided by an individual who did not have any formal financial or educational credentials. Previous studies have demonstrated the effectiveness of teaching from financial professionals in a 10-hour curriculum [17]. Other studies showed benefits dedicated up to 90 minutes for a single subject alone [22] or education that came from physicians dually trained in financial management [14]. The unique circumstances of a single resident educator with a passion for financial literacy severely limit the generalizability of the study findings. Much like in medicine, residents do not need a perfect educator; they just need someone who knows the basics well enough to feel comfortable teaching and answering questions. Beyond the education provided, there is no benchmark for external validity. The questions in the quiz are not gold-standard questions to assess knowledge and were simply pulled from the lecture material - they may have been a more accurate marker of attentiveness in lectures than pertinent financial literacy [18]. 

Follow-up studies are certainly recommended considering these results - primarily to assess the efficacy of short education courses on outcomes. No comparison studies were available to assess one-hour teaching courses that may prove similarly effective. This study does not assess any longitudinal markers of financial success that would be the most appropriate measure of success. Further study could evaluate the difference in net worth in institutions with required financial education and those without to show benefit.

## Conclusions

Residents are at a critical point of education and benefit from a dedicated financial literacy curriculum. It is not evident that residents gain financial knowledge passively during their years of training. Programs can institute dedicated financial literacy education for trainees to improve all-around financial literacy, regardless of the training year. An appropriate financial education curriculum can be instituted without financial professional expertise or prolonged hours of didactics. Instituting these programs have a direct impact on financial independence milestones, and lectures on targeted categories lead to early changes in financial behavior, which will help mitigate the rising costs of medical education.

## Appendices

Financial education quiz

1.       Physicians accumulate wealth faster than their average peers with similar incomes:

a.       True

b.       False

2.       The utility of the paycheck you receive that is most predictive of future financial success would be:

a.       First paycheck ever

b.       First resident paycheck

c.       First attending paycheck

d.       Last attending paycheck

3.       What is the average student loan burden of graduating medical students in 2020?

a.       $180,000

b.       $200,000

c.       $220,000

d.       $240,000

4.       What is the average EM attending physician compensation as of 2020?

a.       $300,000

b.       $350,000

c.       $400,000

d.       $450,000

5.       A mark of indebtedness extended to the owner for compensation in the future with minimal fluctuation in a volatile market is a ____:

a.       Call

b.       Put

c.       Mutual Fund

d.       Bond

6.       In terms of Morningstar evaluations of a company (Small/Large/Growth/Value), companies like Apple and Google would be:

a.       Large Cap Growth

b.       Large Cap Value

c.       Large Cap Blend

d.       Mid Cap Blend

7.       What is the average interest rate on US savings accounts in 2020?

a.       .05%

b.       .5%

c.       1%

d.       2%

8.       Over a long investing horizon, what general relationship does risk have with expected returns?

a.       Risk and Returns are correlated

b.       Risk and Returns are inversely correlated

c.       Risk and Returns are not reliably associated over a long-term period

9.       The biggest advantage of REPAYE over PAYE is:

a.       It qualifies for public student loan forgiveness

b.       It takes into account only the individual’s income instead of the family’s income

c.       You can make Roth contributions

d.       Half of the student loan interest is subsidized

10.   Of all the large student loan processing agencies, which one can directly process PSLF payments with the government as of 2021?

a.       Great Lakes

b.       FedLoan Servicing

c.       Navient

d.       All of the Above

e.       None of the Above

11.   The default payment plan for residents (without extenuating circumstances) should be:

a.       REPAYE

b.       PAYE

c.       Refinance

d.       IDR

12.   If you have $450,000 in student loans at a 6% interest rate and are able to successfully complete the 120 payments for PSLF on REPAYE, you will save how much money compared to IDR:

a.       $200,000

b.       $300,000

c.       $400,000

d.       $500,000

13.   Once you refinance your government loans with a private company (SoFi, Laurel Road, etc):

a.       Your number of qualifying payments toward PSLF resets to 0

b.       You no longer qualify for PSLF

c.       You must send a written statement to your previous loan servicer requesting a payment record transfer

d.       Your PSLF contributions will automatically carry-over

14.   In general, it makes more sense to make ____ retirement fund contributions during your low earnings years and _____ retirement fund contributions during your high earnings years.

a.       Roth, Roth

b.       Roth, Traditional

c.       Traditional, Roth

d.       Traditional, Traditional

15.   How much money can you contribute to your individual retirement account (IRA) in 2021?

a.       $6000

b.       $7000

c.       $8000

d.       $9000

16.   401(k) plans most often:

a.       Are associated with government-backed retirement accounts and are very conservative

b.       Does not have early withdrawal fees or catch-up contributions

c.       Apply to SEP IRAs only

d.       Have a yearly employee contribution limit of $19,500 in 2021

17.   A triple tax-advantaged account that must be applied to healthcare expenditures and grows in a tax-advantaged fashion with yearly contributions:

a.       HSA

b.       529

c.       403(b)

d.       401(a)

18.   A tax-advantaged account (state-by-state variations) for the express purpose of education expenses and transferable between people with no contribution limit is:

a.       HSA

b.       529

c.       Certificate of Deposit

d.       UGMA/UTMA

19.   A tax-advantaged account for the express purpose of cash endowment to a minor between the ages of 18-21 with no defined spending requirements:

a.       HYSA

b.       UGMA/UTMA

c.       Certificate of Deposit

d.       Cash Balance Plan

20.   “If I make enough money to be pushed into another tax bracket, my entire income is now taxed at the new marginal tax rate:”

a.       True

b.       False

21.   Does contributing to a Roth IRA or 401k modify my adjusted gross income (AGI) and does what to my income-driven repayment plan for student loans?

a.       It does not change it

b.       It reduces the required payment

c.       It increases the required payment

22.   Cost Basis is:

a.       The price an investment sold for

b.       The cost/fee associated with selling an investment

c.       The percentage of company profit investment is based on

d.       The price of an investment at the time of purchase

23.   “Riders” on a disability insurance policy are:

a.       Added provisions that modify coverage and increase utility

b.       Is an under-writing process that highlights health concerns

c.       Are restrictions in the event of recreational injury that would exclude coverage

d.       A system of checks and balances to make sure no one branch of government is stronger than another

24.   The difference between whole life/universal life/variable life insurance and term life insurance is that:

a.       Whole life insurance is a commodity and is cheaper to own

b.       Term life insurance accrues interest on your principle

c.       Whole life insurance policies are optimized by over-lapping them

d.       Term life policies are designed to be titrated to financial independence

25.   Umbrella insurance policies are designed to:

a.       Supplement homeowners’ insurance in the event of a third-party injury on your property

b.       Assist that above policy auto-insurance claims after motor vehicle collisions

c.       Be purchased cheaply for low premiums by most home/auto insurance providers

d.       All of the above

26.   What percentage of a typical investor’s portfolio is dedicated to real estate investments in one way or another?

a.       2%

b.       10%

c.       25%

d.       40%

27.   Amortization is:

a.       A calculated table to account for interest and principal paid off at a constant value

b.       A sequence of returns distributed to investors based on their initial investment plus dividends from rent

c.       Insurance the bank charges on mortgages with inadequate down payments to protect their investment

d.       Love potion from Harry Potter

28.   In general, which of these options for real estate investing is the most hands-off, the lowest barrier to entry, and passive investment?

a.       Single dwelling ownership you rent out

b.       REIT’s

c.       Hard money loans

d.       Real estate syndications

29.   What areas of personal finance are you the least familiar or uncomfortable with?

30.   What areas of personal finance are you most interested in learning about?

31.   Which of the following financial milestones have you already accomplished?

a.       Established an IRA

b.       Paid into a 401(k)

c.       Purchased Disability Insurance

d.       Created a personalized financial plan with net worth goals

e.       Created an emergency fund of three months’ salary/six months' expenses

f.        Created a plan for your student loans (PAYE/REPAYE/Refinance)

g.       Paid off credit cards (high-interest debt)

h.       Purchased term life insurance

i.         Opened a personal brokerage account

j.         Purchased a house

k.       Paid off your student loans
